# The role of amino acids in skeletal muscle health and sarcopenia: A narrative review

**DOI:** 10.7555/JBR.38.20240167

**Published:** 2024-10-22

**Authors:** Ramendu Hom Chaudhuri

**Affiliations:** Department of Orthopaedics, Sri Aurobindo Seva Kendra, Kolkata, West Bengal 700068, India

**Keywords:** essential amino acids, skeletal muscle, protein synthesis, sarcopenia.

## Abstract

The skeletal muscle is the largest organ present in the body and is responsible for mechanical activities like maintaining posture, movement, respiratory function, and support for the health and functioning of other systems of the body. Skeletal muscle atrophy is a condition characterized by a reduction in muscle size, strength, and activity, which leads to an increased dependency on others for movement, an increased risk of falls, and a reduced quality of life. Various conditions like osteoarthritis, osteoporosis, and fractures are directly associated with increased muscle atrophy. Additionally, numerous risk factors, like aging, malnutrition, physical inactivity, and certain disease conditions, through distinct pathways, negatively affect skeletal muscle health and lead to muscle atrophy. Among various determinants of overall muscle health, the rate of muscle protein synthesis and degradation is an important parameter that eventually alters the fate of overall muscle health. In conditions of excessive skeletal muscle atrophy, including sarcopenia, the rate of muscle protein degradation usually exceeds the rate of protein synthesis. The availability of amino acids in the systemic circulation is a crucial step in muscle protein synthesis. The current review aims to consolidate the existing evidence on amino acids, highlight their mechanisms of action, and assess their roles and effectiveness in enhancing skeletal muscle health.

## Introduction

Although the bones are the rigid framework that provides the necessary structural stability and posture for the body, they lack the ability to initiate and/or maintain the movement of any body part. Skeletal muscles are associated with the skeletal system and are responsible for body movement. The rhythmic contraction and relaxation of skeletal muscles generate adequate kinetic energy that is essential for the initiation of any voluntary and (in some cases) involuntary movements of the body. The skeletal muscle is the largest organ in the body and comprises approximately 40%–50% of an adult's total body weight^[1–2]^. Skeletal muscle plays a vital role in providing the mass and strength needed for maintaining posture, initiating and maintaining bodily movements, and respiratory functioning^[2]^.

Muscle protein synthesis (MPS) is a highly complex process that involves various molecular pathways. The availability of amino acids (AAs) in the systemic circulation is also crucial for optimal MPS. It is critical to understand the exact pathways by which AAs modulate MPS, with a particular emphasis on muscle degenerative conditions like sarcopenia. The current review aims to consolidate the available evidence on the role of AAs in skeletal muscle health and to highlight the mechanisms by which AAs promote MPS and prevent muscle protein degradation.

## AAs: Introduction and role in the human body

AAs are small organic compounds that are the building blocks for all proteins in the body. Structurally, AAs are compounds that have an amine and carboxylic acid functional group with a carbon skeleton. Various compounds that exist in nature can be identified as AAs based on their structure, but currently, 20 AAs have been identified as being involved in the protein synthesis process in humans^[3]^. Other than protein synthesis, AAs play an important role in the production of various physiologically important substances, including polyamines, glutathione, nitric oxide (NO), and various hormones^[4]^. AAs also have key roles in various cellular signaling, immune system activity, mood and sleep, metabolism, and osmoregulation^[5]^.

Various methods are used for classifying these protein-forming AAs based on their structure, function, and other properties. The most widely used classification method divides these AAs into two types based on their availability, namely essential and non-essential amino acids. Essential amino acids (EAAs) are the AAs that are required to be taken from external or dietary sources, because the body is not able to synthesize them, while non-essential amino acids (NEAAs) are the AAs that are not generally required to be taken from external or dietary sources as the body can synthesize them. Out of the 20 AAs, there are nine EAAs (*i.e.*, lysine, leucine, isoleucine, methionine, valine, threonine, phenylalanine, histidine, and tryptophan) and eleven NEAAs (*i.e.*, arginine, alanine, aspartate, asparagine, cysteine, glutamine, glutamate, serine, proline, glycine, and tyrosine)^[6–7]^. In certain disease conditions, the metabolism of AAs in the body is altered, which increases the requirement for certain AAs that can be either essential or non-essential. Such AAs are termed conditionally essential amino acids (CEAAs)^[8–9]^. In such conditions, the availability of CEAAs becomes necessary to cope with an increased demand by the body, which, if not met, would lead to a CEAA-deficient state that would directly affect various physiological pathways, with the major one being the protein synthesis pathway.

## Role of AAs in skeletal muscle health

Because AAs are structural blocks for protein synthesis and the skeletal muscle is largely composed of proteins, AAs play a crucial role in maintaining normal muscle health and are also important for muscle growth, repair, and overall functioning^[10]^. Among the various determinants of MPS, the presence of optimal AA levels in the systemic circulation is an important parameter that has a strong effect on the fate of the MPS rate.

The mammalian target of rapamycin (mTOR) is a kinase molecule present in the cellular cytoplasm. mTOR is considered a master regulator of protein translation activity and is present in two subunits, namely, mTORC1 and mTORC2^[11–12]^. AAs, particularly leucine and arginine, play a crucial role in directly stimulating the mTOR signaling, thereby initiating the downstream signaling pathways and ultimately causing a spike in the cellular protein synthesis cascade. The presence of AAs activates Rag GTPase to form heterodimers of GTP-bound RagA/B and GDP-bound RagC/D; inversely, in their absence, they form GDP-bound RagA/B and GTP-bound RagC/D. Acting as an upstream regulator of Rag GTPases, the complexes of GTPase-activating proteins towards Rags (GATOR) control the mTORC1 signaling. GATOR1, a subcomplex of GATOR, negatively regulates the activation of Rag GTPase in the absence of AAs^[13]^.

**Figure 1:**
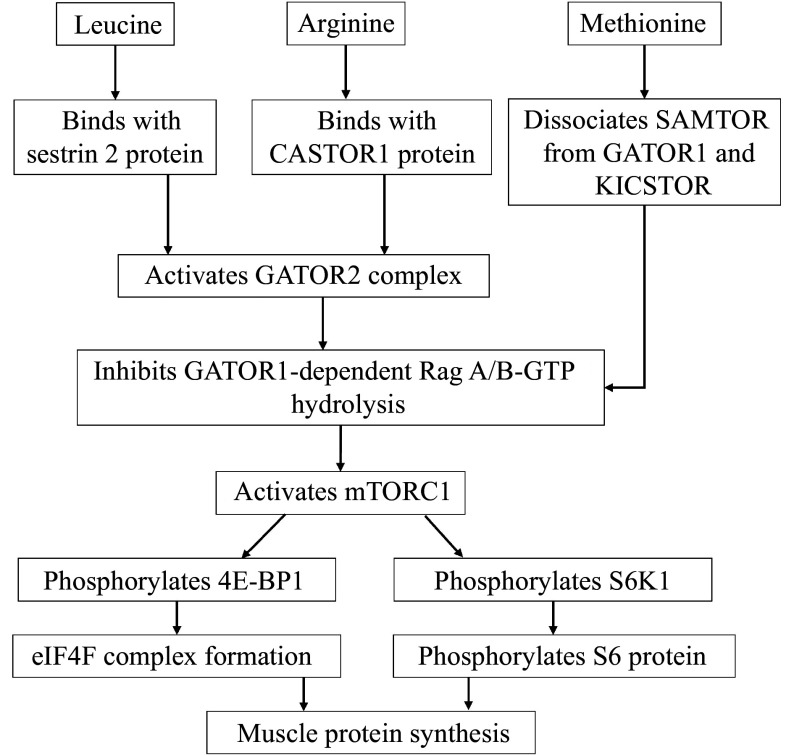
Molecular mechanisms of mTOR activation and muscle protein synthesis. Abbreviations: mTORC1, mammalian target of rapamycin complex 1; CASTOR1, cytosolic arginine sensor for mTORC1 subunit 1; GATOR1/2, GTPase-activating proteins towards Rag 1/2; 4E-BP1, eukaryotic translation initiation factor 4E-binding protein 1; eIF4F, eukaryotic initiation factor 4F; S6K1, ribosomal protein S6 kinase beta-1; SAMTOR, sensor of S-adenosylmethionine.

Leucine plays a role in mTORC1 activation through the sestrin 2 protein. Sestrin 2 acts as a leucine sensor. Under leucine deprivation, sestrin 2 binds to and inhibits the GATOR2 complex, which enables GATOR1 to hydrolyze the GTP-RagA/B complex, preventing the recruitment of mTORC1 to the lysosome and thereby inhibiting mTORC1 activation. When leucine levels are restored, leucine binds to sestrin 2, causing sestrin 2 to dissociate from GATOR2. This dissociation relieves the inhibition of mTORC1, allowing it to be recruited to the lysosome and activated. Additionally, arginine activates mTORC1 through the cytosolic arginine sensor for mTORC1 subunit 1 (CASTOR1), a cytosolic arginine sensor. In the absence of arginine, CASTOR1 inhibits GATOR2 by binding to it, which prevents mTORC1 activation. Upon arginine availability, arginine binds to CASTOR1, causing it to dissociate from GATOR2, which allows mTORC1 to be activated. Another important regulator of mTORC1 activity is the sensor of S-adenosylmethionine (SAMTOR). In the absence of methionine, SAMTOR activates GATOR1 *via* the KPTN, ITFG2, C12orf66, and SZT2-containing regulators of mTOR (KICSTOR). In the presence of an adequate methionine level, the activity of SAMTOR is reduced, thereby inhibiting the activity of KICSTOR and GATOR1, and therefore stimulating mTORC1 activity^[14–16]^. The activation of mTORC1 initiates protein synthesis through two major mechanisms: (a) phosphorylating and inhibiting the eukaryotic translation initiation factor 4E (eIF4E)-binding protein 1 (4E-BP1), thus releasing the eIF4E to form a complex with eukaryotic initiation factor 4F (eIF4F) and thereby initiating ribosome recruitment and the protein translational process; and (b) phosphorylating the ribosomal S6 protein by stimulating the ribosomal protein S6 kinase beta-1 (S6K1), an essential step required for translation (***Fig. 1***)^[17]^.

Some evidence has also suggested the role of AAs in activating pancreatic β-cells, which subsequently improves the insulin secretion rate. As skeletal muscles are primarily responsible for generating the force required for any locomotor activity, they are also the site in the body that utilizes the highest amount of glucose to maintain an adequate energy level. Insulin is the major hormone in the body and is responsible for the transfer of glucose from the systemic circulation into skeletal muscle, which thereby improves the energy level. Moreover, insulin is suggested to increase the activation of mTOR within skeletal muscles, both directly and indirectly by reducing AMPK levels. Because of this dual role, insulin is considered an anabolic hormone that has protein synthesis-stimulating activity in skeletal muscles^[11–12]^. Hence, by directly incorporating into the cytoskeletal proteins and by activating pathways responsible for initiating cellular protein synthesis, AAs play an important role in skeletal muscle growth, development, and overall health^[18–19]^.

In the context of skeletal muscle health, both EAAs and NEAAs play pivotal roles in MPS. However, the effectiveness of specific AAs, either individually or in combination, varies depending on their metabolic roles and mechanisms of action. Branched-chain amino acids (BCAAs), particularly leucine, are potent stimulators of MPS, particularly in elderly populations and those suffering from sarcopenia. However, while BCAAs alone can enhance MPS, studies suggest that a complete EAA mixture is more effective in sustaining MPS over time^[20–21]^. For instance, a study found that older adults experienced a more significant increase in MPS when consuming a high proportion of leucine-enriched EAAs, compared with those who consumed leucine alone, suggesting that the proportion of leucine within an EAA mixture is critical for maximizing the anabolic response, especially in populations at risk of muscle atrophy, such as those with sarcopenia^[20]^. Similarly, in another clinical study, it was observed that participants supplemented with a combination of EAAs with intermediates of the tricarboxylic acid cycle showed better muscle health parameters, compared with those who supplemented with BCAAs alone^[21]^. Such observations underscore the need for all EAAs to achieve optimal beneficial effects on improving muscle health.

## Sarcopenia: Introduction and consequences

Sarcopenia is a skeletal muscle disease characterized by progressive and generalized loss of muscle mass, strength, and associated functionality. The European Working Group on Sarcopenia in Older People (EWGSOP) has classified sarcopenia into two types, namely primary and secondary sarcopenia. Primary sarcopenia is characterized by muscle loss because of aging, whereas secondary sarcopenia is characterized by muscle loss because of factors other than aging, including bed rest, a sedentary lifestyle, inadequate nutrition, smoking, chronic disease conditions (*e.g.*, metabolic syndrome, depression, Parkinson's disease, anorexia, anemia, osteoporosis), and certain medications^[22–23]^. The worldwide prevalence of sarcopenia is estimated to be 10% to 27% in the elderly population^[24]^, and sarcopenia is more prevalent in patients than in healthy populations^[23]^.

**Figure 2:**
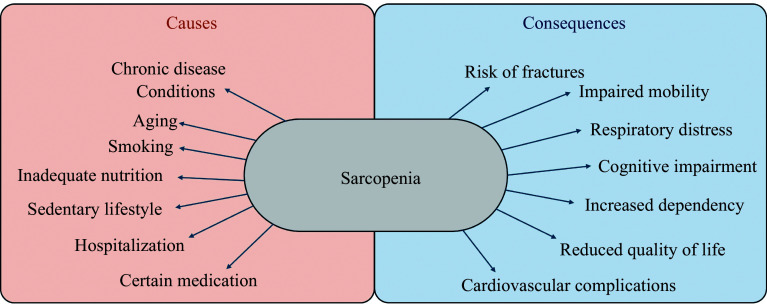
Causes and consequences of sarcopenia.

Individuals with sarcopenia are highly vulnerable to adverse personal, social, and economic consequences. The personal health consequences of sarcopenia include an increased risk of falls and fractures, impaired activities of daily living, cardiovascular complications, respiratory distress, mental and cognitive impairment, impaired mobility, increased dependency and loss of independence, as well as a significantly negative influence on the overall quality of life. Additionally, sarcopenia increases the healthcare costs of the individual by increasing the hospitalization rate and care needs during the hospitalization period (***Fig. 2***). Data from various community-based studies have shown that the hospitalization costs of individuals with sarcopenia are five times more likely to be high, compared with those without sarcopenia. These data are consistent in numerous studies, irrespective of the community setting and age of the participants^[25]^.

## Sarcopenia: Pathophysiology

**Figure 3:**
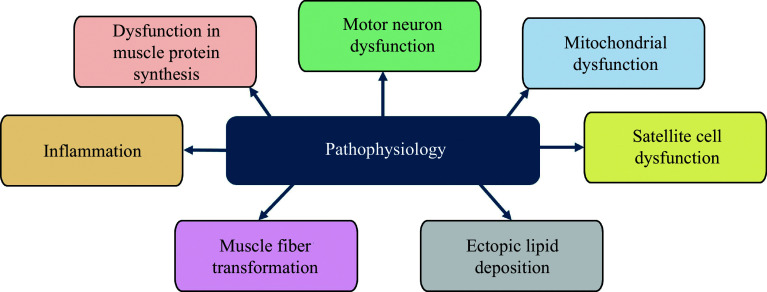
Pathophysiology of sarcopenia.

The research on sarcopenia has increased substantially in the recent decade, with some evidence suggesting that sarcopenia is a complex pathological condition involving various simultaneously acting pathways, including satellite cell abnormality, alterations in the MPS pathway, the biotransformation of muscle fibers, mitochondrial dysfunction, increased reactive oxygen species, elevated fat deposition, impaired motor-neuron activity, and chronic systemic inflammation (***Fig. 3***).

### Satellite cell dysfunction

The muscle repair and regeneration processes primarily rely on the activation of satellite cells. After muscle damage, satellite cells are activated, enter the cell cycle, proliferate into myogenic precursor cells, and further differentiate to fuse with existing muscle fibers or form new ones to completely heal the damaged muscle site^[26]^. In aging and chronic disease conditions, factors such as reduced antioxidant capacity, increased DNA damage, and altered gene expression levels cause a progressive reduction in satellite cell number and activity. This decline in satellite cell number and activity is correlated with an impaired muscle regenerative potential, leading to sarcopenia^[27–28]^.

### Dysfunction in muscle protein synthesis

Among the various determinants of MPS, the major parameters include activation of the mTOR signaling pathway, adequate functioning of insulin in skeletal muscle, and sufficient cellular energy levels in skeletal muscle. These parameters have a direct and most potent influence on the overall MPS^[29]^. However, with aging, the sensitivity of mTOR is observed to be reduced, probably because of the prolonged and sustained activation of mTOR, which is correlated with muscle atrophy^[30]^. Additionally, in disease conditions that involve impaired insulin sensitivity and increased insulin resistance, the activity of insulin at the skeletal muscle level is altered, reducing the skeletal muscle glucose intake, glycogenic pathways, and muscle cell energy substrate (ATP) levels. The reduction in cellular ATP levels is further correlated with a reduced ATP/AMP ratio, which leads to the AMPK activation and subsequently inhibits the mTOR signaling cascade, further suppressing the MPS pathway^[11]^.

Other than the impaired MPS, sarcopenia is correlated with increased muscle protein degradation. Various pathways, including autophagy, the ubiquitin proteasomal system, and calpain-related signaling pathways, have been identified to play an important role in skeletal muscle protein degradation^[31]^. These reduced MPS and increased degradation rate collectively result in a net skeletal muscle catabolic state, ultimately causing a reduction in muscle size and strength.

### Muscle fiber transformation

Based on their functionality, skeletal muscle fibers are classified into two types: slow-twitch (type 1) muscle fibers and fast-twitch (type 2) muscle fibers^[32]^. During muscle aging, senescent muscles undergo significant alterations at the cellular and molecular level, including a change in muscle fiber activation rate, a reduction in excitation-contraction coupling activity, an altered actin-myosin cross-linking, and an alteration in energy production rate^[33]^. Because of these alterations, the muscle filaments undergo a characteristic transformation from fast-to-slow twitch muscle fibers, which is reflected by a reduction in fast myosin heavy chain isoforms (MyHC-2a and MyHC-2x) and an increase in slow myosin heavy chain isoform (MyHC-1)^[34]^. Such alterations cause the molecular and functional switch from type-2 to type-1 muscle fibers, which is characteristically observed in the sarcopenic condition. This functional switch of muscle fibers reduces the activation speed, response time, and functionality of muscles, resulting in increased stiffness, fatigue, and reduced functional capability in individuals with sarcopenia.

### Increases in reactive oxygen species and mitochondrial dysfunction

Mitochondria are important energy-regulating cellular components that produce cellular ATP through the oxidative phosphorylation process^[35]^. The process of energy production within the mitochondria involves a complex chain of reactions, which is termed the respiratory chain process^[36]^. The respiratory chain process is a single-electron exchange reaction process that yields numerous by-products during the entire reactive cascade, with the most important by-product being reactive oxygen species (ROS). ROS are small, molecularly charged, reactive molecules that have numerous signaling functions within the cell^[37]^. ROS are generated by almost all cellular components, and around 90% of cellular ROS are generated by the mitochondria because of their extensive role in utilizing oxygen species to form ATP^[38]^. Besides the production of ROS, mitochondria have a ROS-scavenging pathway as well, which helps maintain an intricate ROS level in the cell^[36,39]^.

Various pieces of evidence have suggested the important roles of ROS for optimal cellular functioning, which include triggering pathways correlated with cellular protection, the initiation of mitochondrial fission, and autophagy reactions to remove any abnormal organelle^[39]^. An imbalance in the controlled ROS production cascade may lead to either under- or over-production of ROS, both of which are correlated with characteristic pathological states. In sarcopenia, data from some evidence have suggested a possible and critical link between high ROS generation and muscle cell death^[40]^. This is because mitochondrial DNA (mtDNA) is around 10–20 times more susceptible to ROS-mediated mutagenesis and damage than nuclear DNA, as mtDNA is in close proximity to the ROS-producing site^[41]^. This alteration of and damage to the mtDNA reduces the oxidative capability of mitochondria and also damages the mitochondrial structure, causing a reduced energy production and initiation of the chain of ROS-induced ROS production and release^[39,41]^. All these alterations cause the activation of apoptotic signaling pathways, increase cellular oxidative stress, and disrupt cellular functioning, leading to cell apoptosis and death. With aging and chronic muscle damage, the increased ROS levels in the skeletal muscle may be directly linked to the described pathway, which is correlated with the reduced mass and strength observed in sarcopenia^[42]^.

### Ectopic lipid deposition

Adipose tissues also serve a dual role as an immune-endocrine organ and an energy storage site. Triglycerides stored in adipose tissues are broken down into free fatty acids and glycerol that are transported for energy use. In aging individuals, impaired adipogenesis reduces the ability of white adipose tissues to buffer free fatty acids. Obesity exacerbates the problem by causing abnormal fat deposits in tissues like the liver, muscle, heart, and pancreas. The excessive ectopic lipids in the skeletal muscle lead to lipotoxicity, characterized by an increase in the release of cytokines, adipokines, and chemokines, ultimately causing muscle wasting and mitochondrial dysfunction^[43]^.

### Motor neuron dysfunction

A motor unit comprises a single alpha motor neuron (α-MN) and the related muscle fibers. In the event of the loss of an α-MN, the related muscle fibers undergo structural changes to connect with adjacent surviving α-MNs. Such an adjustment with the adjacent α-MNs results in the formation of larger motor units, which ultimately contributes to a decline in muscle efficiency, potentially causing the tremors and fatigue commonly observed in elderly individuals^[44]^. It was also noted that after 70 years of age, the number of α-MNs decreases by approximately 50%, leading to a reduced muscle coordination^[29]^.

### Inflammation

Aging is commonly correlated with a chronic systemic low-grade inflammatory state, which is a consequence of various factors including a reduced nutritional intake, hormonal changes, and a decreased physical activity^[45]^. The surge in levels of pro-inflammatory cytokines, including tumor necrosis factor-α (TNF-α) and interleukin-6 (IL-6), is correlated with a negative effect on the growth and metabolic state of skeletal muscles as well as being involved in promoting muscle protein breakdown and impairing the anabolic processes responsible for muscle maintenance and repair. Additionally, the inflammatory environment attenuates the MPS, which in turn disrupts the delicate balance between muscle protein formation and degradation, favoring the latter and leading to a net loss of muscle mass. Moreover, chronic inflammation induces insulin resistance and reduces levels of anabolic hormones, mainly insulin-like growth factor 1 (IGF-1), which further exacerbates muscle protein breakdown. These degradative mechanisms underscore the pathological role of low-grade inflammation in muscle homeostasis^[46–47]^.

## Sarcopenia: Risk factors

### Type 2 diabetes mellitus (T2DM)

T2DM is a chronic metabolic condition characterized by increased systemic glucose levels and insulin resistance. As previously discussed, the skeletal muscle is one of the targets for the insulin action that subsequently leads to MPS *via* the activation of mTOR, raising the possibility that T2DM may have a potential effect on the metabolic state of muscles^[11]^. The surge in glucose levels and insulin resistance is correlated with increased muscle wasting, thereby negatively affecting overall muscle health. Various molecular pathways have been identified in which high glucose levels play a significant role in hyperglycemia-induced muscle degradation, with the key role of the ubiquitin-proteasome pathway (UPP) and WWP1/KLF15 pathway. UPP is an endogenous protein-degrading mechanism that involves the coordinated activity of various proteins and enzymes, leading to the degradation of target proteins *via* the activity of three essential enzymes, namely ubiquitin-activating enzyme (E1 enzyme), ubiquitin-conjugating enzyme (E2 enzyme), and ubiquitin ligase (E3 ligase). These enzymes collectively facilitate the tagging of proteins with ubiquitin, marking them for subsequent degradation by proteasomes into small peptides or AAs^[48]^. WWP1 is an endogenous E3 ligase that is known to prevent muscle atrophy during hyperglycemia by particularly targeting the Krüppel-like factor 15 (KLF15) protein, leading to the UPP-dependent KLF15 degradation. KLF15 is a transcription factor that regulates the metabolisms of carbohydrates, proteins, and lipids. During conditions of insulin resistance, the activity of WWP1 is reduced, which inhibits UPP-dependent KLF15 degradation, thereby increasing the levels of KLF15. These increased KLF15 levels in muscle cells further lead to the upregulation of genes that are related to muscle atrophy^[49]^. Additionally, insulin resistance leads to the activation of other UPP enzymes that particularly target the MPS-related genes and signaling factors, thereby preventing the MPS, which collectively causes an increase in muscle protein degradation and a reduction in muscle protein formation, leading to net muscle catabolism^[50]^. This particular mechanism was confirmed in a streptozotocin-induced diabetic model, in which mice deficient in muscle-specific KLF15 showed protection against hyperglycemia-induced skeletal muscle atrophy, while streptozotocin treatment in wild-type mice showed muscle atrophy^[49]^.

### Osteoarthritis (OA)

OA is a chronic joint degenerative disease that causes progressive destruction of the articular cartilage, synovial membrane, ligaments, and subchondral bone^[51]^. Some clinical evidence has shown an association between OA and reduced lower limb muscle strength^[52–53]^. While the direct correlation between OA and muscle wasting is not well understood, various studies have indicated that OA is correlated with a chronic inflammatory environment and the increased gene expression of muscle-degrading proteins, which leads to muscle degradation. A preclinical study in rats with OA showed that the levels of IL-1β and myostatin increased while the expression levels of myogenin decreased^[54]^. Myostatin is a protein that functions to control the hypertrophy of myoblasts by preventing their proliferation and differentiation rates. Some evidence suggests that an increase in myostatin level is correlated with muscle wasting, because it inhibits the growth of muscle cells and also promotes the activation of UPP^[55]^. Myogenin, on the other hand, is a transcription factor specific to skeletal muscle that is involved in the induction of myogenesis and the increase of skeletal MPS^[55]^. With alterations in the levels of myostatin and myogenin, it may be plausible to say that OA has a direct and negative effect on muscle health and promotes muscle atrophy and sarcopenia. The findings of preclinical studies were further confirmed in real-world clinical settings, where OA patients showed some elevated levels of muscle inflammatory markers, such as monocyte chemotactic protein-1, p65, NF-κB, and IL-6, as well as an increase in the activity of signal transducer and activator of transcription 3, which identified the chronic inflammatory state as playing an important role in the muscle atrophy condition^[55]^. Furthermore, patients with moderate knee OA showed a lower density of satellite cells, indicating some impaired muscle regenerative capacity, and high expression of the profibrotic gene, suggesting an increased fibrosis, which leads to the reduction in muscle quality^[53]^.

### Bone fracture

A bone fracture is one of the most common orthopedic injuries, characterized by a break or discontinuity in the bone tissues^[56]^. Patients with hip fractures have shown a negative impact on their muscle strength^[57]^, and patients with vertebral fractures have shown significantly reduced hand grip strength, leg extension, arm curl, sit-to-stand test, and step test^[58]^. In pelvic ring fracture patients, hip muscle strength was significantly affected, compared with the controls^[59]^. In all cases, the primary reason for muscle loss is a reduction in physical activity after a fracture, which is because of the restriction of movements caused by the increased pain^[60]^.

### Surgery-induced muscle loss

Various surgical procedures lead to a rise in inflammatory markers and oxidative stress, a muscle disuse, and a reduced protein intake, which collectively cause muscle wasting^[61]^. For instance, the patients who underwent total knee arthroplasty showed slower walking speed, longer stair climbing time, and lower knee extension, compared with the control subjects^[62]^. Furthermore, the quadriceps and hamstring muscle thickness were significantly reduced at six weeks after total knee replacement (TKR)^[63]^. The possible reasons for the muscle loss may be the increases in catabolic activity and muscle proteolysis, which are accelerated as a result of the surgical procedure^[64]^. In hospitalized patients, elevated levels of myostatin mRNA, which drives muscle atrophy, and suppression of IGF-1, which promotes the hypertrophy of muscle mass, were observed^[65]^.

## Available therapeutic options

While various risk factors and molecular targets have been identified for sarcopenia, a very small number of treatment options are currently available. The most promising therapy is resistance training and/or exercise. Various pieces of clinical evidence have supported the positive role of exercise in improving muscle strength and functional performance^[66]^. Other than resistance exercise, protein supplements are also used as a treatment option. However, the major complication with protein supplementation is the huge variation in clinical benefits because of different protein supplements. As it is evidenced that different protein supplements have different clinical benefits, the generalization of clinical study results to all available protein supplements should be avoided. Such generalization of any clinical study results to a real-world setting may cause detrimental effects on individuals with sarcopenia by increasing their healthcare expenditure and therapy burden, while providing no additional clinical benefits to overall muscle health^[67]^.

Supplementation of AAs is also used in clinical settings to treat sarcopenia. This is based on the fundamental understanding that AAs are the structural units of proteins and have a better absorption profile than whole protein. Results from various clinical studies support the notion that plasma AA concentration and MPS rate are significantly higher in the AA supplements plus whey protein group than in the whey protein alone supplementation group^[68]^. Additionally, AA supplementation has been shown to improve muscle mass and strength and reduce inflammatory markers^[69–70]^.

The use of antioxidants, such as vitamin C and vitamin E, is also justified based on the fact that oxidative stress plays an important role in muscle wasting, and antioxidants may help prevent this oxidative stress-induced muscle wasting^[71]^. Furthermore, vitamin D deficiency has shown an association with muscle loss and muscle quality, indicating that vitamin D supplementation may effectively prevent muscle loss in sarcopenia, supported by a clinical trial in which vitamin D supplementation demonstrated an improvement in muscle strength^[72–73]^.

## Amino acids, muscle health, and sarcopenia

As AAs play a crucial role in MPS, there are certain AAs that become essential in sarcopenia because of either the increased demand for AAs in MPS, a reduced ability of the body to endogenously produce AAs, or both. Such a condition causes a net negative balance in the level of such AAs, and hence these AAs are then classified as CEAAs, and the exogenous supplementation of these AAs becomes utmost necessary for preventing muscle protein degradation and improving MPS. While all 20 AAs have a specific role in the body, the roles of leucine, lysine, arginine, valine, methionine, isoleucine, phenylalanine, threonine, histidine, and tryptophan are widely studied and accepted as important for skeletal muscle health.

Leucine plays a primary role in activating the mTOR activity in various tissues, including skeletal muscles^[74]^. Because of the leucine's direct mTOR-stimulatory effect, the intake of leucine was correlated with an increase in the number of satellite cells and their activation in skeletal muscles^[75]^. These observations were supported by the result of a clinical study in which leucine supplementation in a sarcopenia patient resulted in an improvement in muscle mass, walking speed, and knee extension^[76]^. Similarly, lysine plays an important role in the activation and proliferation of satellite cells in skeletal muscle, *via* activating the mTORC1 signaling pathway^[77]^. Arginine, being a biological precursor of NO, is also important for maintaining optimal muscle health. NO is an endogenous signaling molecule that is released from the endothelial lining and causes vasodilation, reduces platelet aggregation, and inhibits mast cell-induced inflammation^[78]^. NO production in skeletal muscle is associated with improved metabolic functions, such as blood flow, glucose uptake, and oxidative phosphorylation, resulting in increased energy levels^[78]^. Hence, it is hypothesized that arginine supplementation in sarcopenia improves overall blood flow and metabolic functioning in skeletal muscles, thereby improving overall muscle health. Methionine is the major AA involved in the endogenous synthesis of glutathione, a potent antioxidant that helps counter oxidative stress^[79]^. As the deleterious role of oxidative stress in muscle health is well-defined, methionine supplementation in sarcopenia may improve overall antioxidant capacity, thereby reducing oxidative stress and related muscle wasting.

Leucine, isoleucine, and valine are widely known as BCAAs based on their distinct structures. Other than leucine, the other BCAAs, namely isoleucine and valine, play a crucial role in activating the malate-aspartate shuttle pathway in the muscles. The malate-aspartate shuttle pathway is important for maintaining cellular and mitochondrial redox potential, which is thereby essential for the oxidative phosphorylation-related ATP production in the mitochondria^[80–81]^. An impairment in the malate-aspartate shuttle pathway may drastically alter the redox potential and thereby reduce the muscle energy level^[82]^. In older patients with sarcopenia, lower levels of histidine and tryptophan were observed, which are the key AAs required for the proper functioning of muscles^[83–85]^. Moreover, threonine and phenylalanine help with skeletal MPS by activating IGF-1^[86–87]^.

## Amino acids: Molecular effect in skeletal muscle

**Figure 4:**
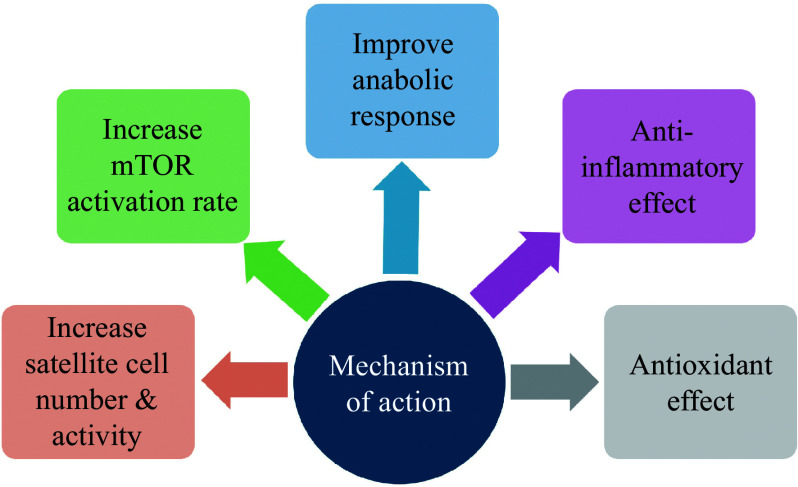
Mechanism of action of amino acid supplementation in muscle health. Abbreviation: mTOR, mammalian target of rapamycin.

Various pieces of evidence have suggested that AAs are involved in protein synthesis, metabolism, and the regulation of signaling pathways in skeletal muscle. Other than being the structural unit for protein synthesis, AA supplementation is associated with an increase in the number and activation rate of satellite cells, an improvement in the mTOR activation rate, an increase in the level and activity of anabolic hormones and molecules, an anti-inflammatory effect, and an antioxidant effect (***Fig. 4***).

### Satellite cell activation

Satellite cells are essential precursor stem cells of skeletal muscles, and when activated, they play a crucial role in the regeneration of injured muscle fibers^[1]^. A clinical study underscored that during conditions of increased muscle wasting, the number of satellite cells was significantly reduced, but the supplementation of AAs significantly improved the number and activation rate of satellite cells^[88]^. The study included patients undergoing TKR surgery, and they were supplemented with the combination of histidine, isoleucine, leucine, lysine, methionine, phenylalanine, threonine, and valine. After the surgery, it was observed that patients supplemented with AAs had a significantly greater number of satellite cells and a significantly improved activation rate, compared with patients who were not treated with AAs, suggesting that the optimal availability of AAs is crucial for the activity of satellite cells^[88]^. While this study identified the anti-inflammatory effect of the supplemented AAs responsible for their roles in satellite cells, future studies are required to confirm these findings^[88]^.

### mTOR activation

As discussed earlier, the mTOR pathway is a key regulator of MPS. mTORC1, an mTOR complex, is recognized as a crucial regulator in the control of skeletal muscle mass. Its role extends to various processes, including contraction and mechanical load-induced hypertrophy, synergistic ablation, myotube hypertrophy, and AA sensing^[89]^. Some preclinical studies found that the treatment of human fibroblasts with AAs was correlated with reduced protein degradation through distinct signaling pathways^[90]^. AAs, such as arginine, leucine, and methionine, potently activate mTORC1, promoting anabolism by phosphorylating translation-related proteins like S6K1 and 4E-BP1 to enhance protein synthesis^[91]^.

### Improve anabolic response

Muscle protein anabolism is very well controlled by upstream triggers, such as IGF-1 and intermediary proteins, which activate the mTOR pathway^[92]^. As IGF-1 binds to its receptor, the cascade starts and phosphorylates p70, which ultimately leads to protein synthesis^[93]^. MPS and muscle protein breakdown are simultaneous processes, occurring in harmony in healthy muscle. To increase muscle mass, the anabolism of protein in muscle should exceed the muscle protein breakdown^[94]^. Any imbalance in this homeostasis may result in muscle atrophy, leading to a reduction in muscle strength^[94]^. A study showed that after exercise, AA supplementation increased MPS as well as reduced muscle protein breakdown, concluding that AA supplementation improves the anabolic response of MPS^[95]^.

### Anti-inflammatory and antioxidant effects

Aging increases chronic inflammation, which is associated with muscle catabolism as the body is not efficiently producing the required energy^[45,96]^. Along with that, the imbalance in ROS production leads to increased ROS levels and ultimately causes damage to muscle cells^[40]^. AAs, such as histidine, lysine, and arginine, exhibit strong antioxidant and anti-inflammatory activities^[97–98]^. Methionine also plays an important role in immune regulation, as its catabolism yields an increased production of glutathione, a known antioxidant, along with other metabolites^[79]^.

## Clinical evidence

A randomized placebo-controlled study included 60 patients with TKR for knee OA^[64]^. Patients were randomized to receive either a blend containing ten AAs, which included nine EAAs (*i.e.*, lysine, leucine, valine, methionine, isoleucine, phenylalanine, threonine, histidine, and tryptophan), along with arginine, or a placebo lactose powder. The supplementation was carried out one week before the surgery and two weeks after the surgery. The changes in the rectus femoris muscle area and the quadriceps muscle diameter were the primary endpoints considered for the study. Supplementation with AAs was correlated with an increase in the rectus femoris muscle area and the quadriceps muscle diameter, compared with the placebo group, but statistical significance between the groups was not reached (*P* = 0.457 and *P* = 0.861, respectively). However, knee pain after surgery was significantly reduced in the AA supplementation group (*P* = 0.038), compared with that in the placebo group.

A similar study was conducted on 52 patients undergoing unilateral TKR with a two-year follow-up^[99]^. The patients assigned to the treatment group were given a blend containing almost similar AAs as in the previous study, and the placebo group was given lactose powder. The participants consumed the allotted treatment one week before and two weeks after the surgery. At the two-year follow-up, a significant improvement was found in the diameter of the rectus femoris (*P* = 0.009), rectus femoris muscle area (*P* = 0.01), and quadriceps muscle strength (*P* = 0.02) in the supplementation group, compared with the placebo group. These results suggested that AA supplementation plays a beneficial role in muscle mass, even in the long run.

To evaluate the effect of EAAs along with arginine in glucose-intolerant patients, a trial was conducted involving 12 diabetic patients who were supplemented with EAAs along with arginine for 16 weeks, and parameters like lean body mass, lower limb strength, and functionality were evaluated^[100]^. A significant increase in lean body mass (*P* < 0.05), lower limb strength (*P* < 0.001), usual gait speed (*P* = 0.002), timed 5-step test (*P* = 0.007), and timed floor-transfer test (*P* = 0.022) was observed after the AA supplementation. While the results were consistent with previous studies, this study highlighted the safety of EAAs along with arginine supplementation in diabetic patients suffering from muscle wasting. Similarly, another clinical study was conducted to evaluate the effect of AA supplementation on pain levels in elderly patients with hip fractures^[101]^. Forty participants were randomly assigned to receive either the blend of AAs or the placebo maltodextrin for four weeks. After the supplementation, a significant decrease in pain level (*P* < 0.001) was observed in the treatment group, compared with the placebo group.

While various studies have confirmed the beneficial role of AAs in muscle health, many studies have also confirmed the benefits of protein supplementation, especially whole whey protein, in improving overall muscle health^[102]^. Hence, to understand the beneficial effect of individual AA supplementation and whey protein supplementation as a whole in improving MPS rate, a clinical study was conducted to compare the effect of AAs against intact whey protein on MPS^[103]^. The treatment group (*n* = 7) received AA supplements, while the control group (*n* = 8) received a whey protein in an isocaloric amount. The MPS was measured by the mixed muscle fractional synthetic rate (FSR). Results showed that the AA supplementation increased FSR significantly, compared with the whey protein supplementation. The authors concluded that the observed improved FSR was because of faster and higher absorption of AAs in the systemic circulation from AA supplements rather than intact whey protein supplements. The improved absorption rate resulted in a three-fold increase in net FSR (*P* < 0.05), as observed by the improvement in the net phenylalanine uptake rate (*P* < 0.05). Based on these observations, the authors also concluded that for providing equivalent FSR between the AA supplements and intact whey protein supplements, it is essential to supplement with a higher dose (two times higher) of whey protein supplements, compared with AA supplements.

## Concluding remarks

In conclusion, the current review underscores the pivotal role of EAAs in maintaining skeletal muscle health. By investigating their effects on protein synthesis, cellular signaling, and muscle function, it also highlights the significance of AAs in preventing muscle wasting through diverse mechanisms. Additionally, the article determines the roles of factors, such as aging, medical interventions, and metabolism, in muscle health, and discusses potential therapeutic applications of amino acids and nutritional strategies. Both preclinical and clinical evidence indicate that AAs function through multifaceted mechanisms, enhancing muscle protein synthesis, increasing muscle mass, and improving muscle function. These highlight the crucial importance of AAs in promoting optimal muscle health.
